# Supplementary material for: The ammonia oxidizing bacterium Nitrosomonas eutropha blocks T helper 2 cell polarization via the anti-inflammatory cytokine IL-10
Source: Sci Rep. 2021 Jul 8;11:14162. doi: 10.1038/s41598-021-93299-1 (PMC8266879; doi:10.1038/s41598-021-93299-1)
Supplement: Supplementary file 1 — Supplementary Information. [file 41598_2021_93299_MOESM1_ESM.docx]

**The ammonia oxidizing bacterium *Nitrosomonas eutropha* blocks T helper 2 cell polarization via the anti-inflammatory cytokine IL-10**

Damien Maura^1^, Nazik Elmekki^1^, and C. Alex Goddard^1^*

^1^ AOBiome Therapeutics LLC, Cambridge MA

* corresponding author: [projectmanager@aobiome.com](mailto:projectmanager@aobiome.com)

**SUPPLEMENTARY FIGURES**

**
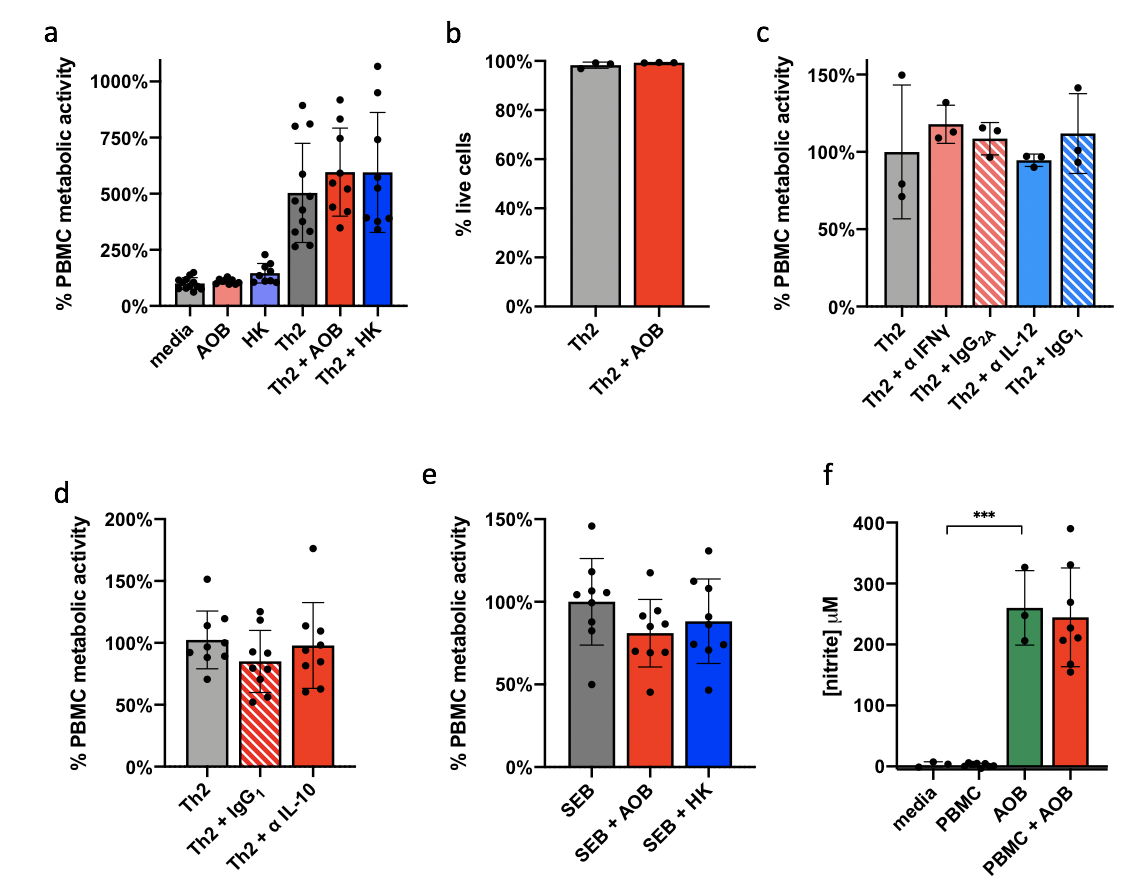
**

**Figure S1 – Various AOB or blocking antibody treatments are not toxic to PBMC, and AOB retain metabolic activity when in contact with PBMC**

(a) PBMC metabolic activity is unaffected by addition of live or heat killed AOB and increased after Th2 stimulation; measured by WST-1 as an indicator of cell viability in samples collected 72h after treatment (n**≥**9, donor A, one-way ANOVA with multiple comparisons). (b) Percentage of live PBMC is unchanged in presence or absence of AOB; measured by LIVE/DEAD staining (n=3, donor C, unpaired t test). (c) PBMC metabolic activity is not significantly different in the presence or absence of IFNɣ or IL-12 neutralizing antibodies or isotype controls; measured by WST-1 (n=3, donor A, one-way ANOVA with multiple comparisons) (d) PBMC metabolic activity is not significantly different in the presence or absence of IL-10 neutralizing antibody or isotype control; measured by WST-1 (n=3 per donor, 3 donors, one-way ANOVA with multiple comparisons). (e) PBMC metabolic activity after SEB stimulus is not significantly different in presence or absence of live or heat killed AOB (n=3 per donor, 3 donors, one-way ANOVA with multiple comparisons). (f) Nitrite is not produced by PBMC alone but is produced by AOB both in presence and absence of PBMC; measured by Griess assay (n**≥**3, one-way ANOVA with multiple comparisons).

**
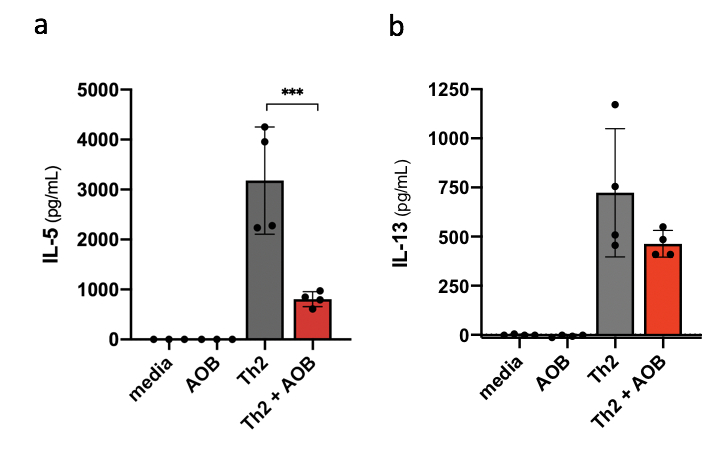
**

**Figure S2 – AOB reduce the production of Th2 cytokines by PBMC collected 5 days post-stimulation**

(a, b) IL-5 (a) and IL-13 (b) production is reduced with AOB pretreatment prior to stimulation by Th2 differentiation cocktail; measured by ELISA from culture supernatants of PBMC collected 5d post-stimulation (n=4, donor C, one-way ANOVA with multiple comparisons).

**
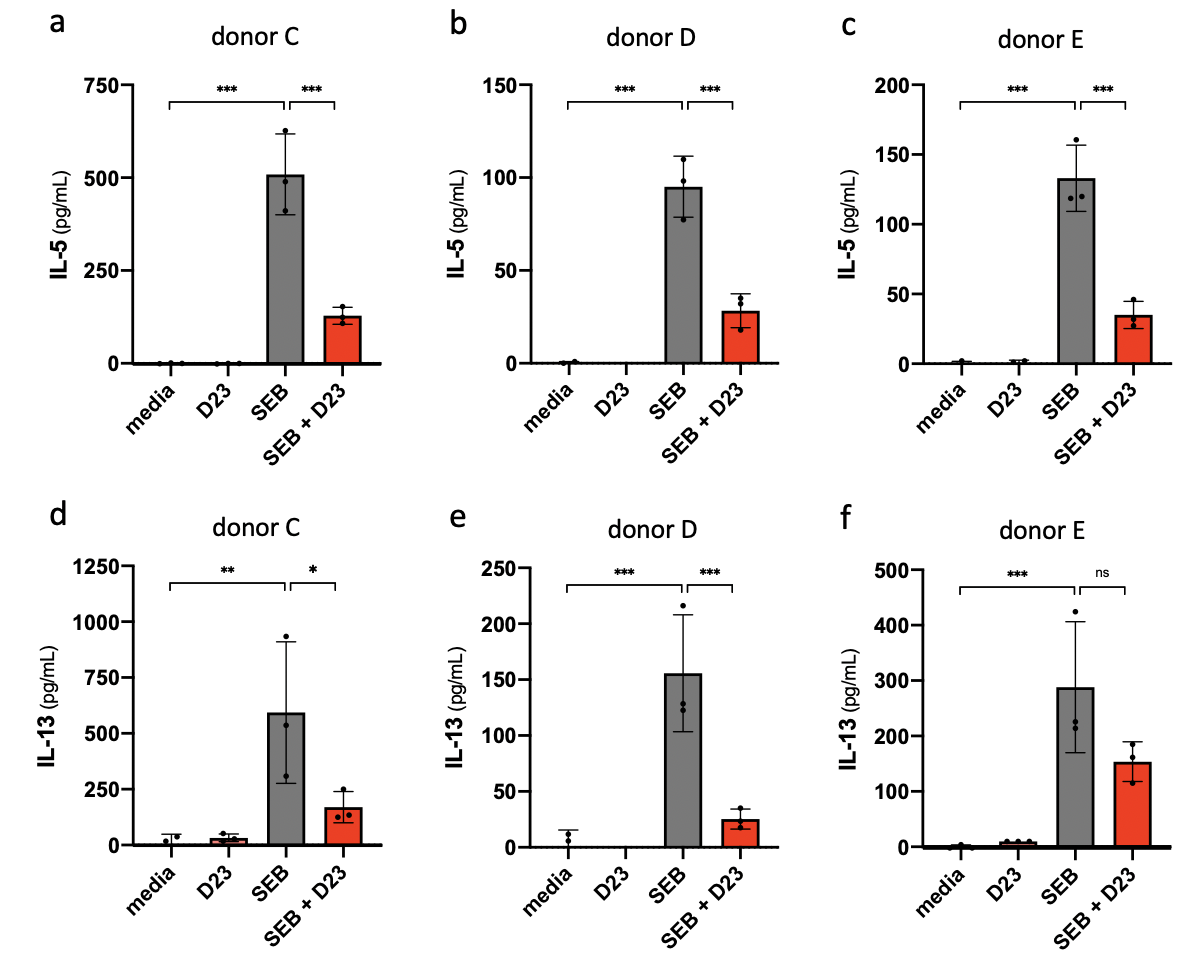
**

**Figure S3 – Th2 cytokines production by PBMC is induced by SEB and reduced by AOB**

IL-5 (a-c) and IL-13 (d-f) production is stimulated by SEB pretreatment and reduced with AOB pretreatment; measured by ELISA from culture supernatants of PBMC collected 7d post-stimulation in donors C (a, d), D (b, e) and E (c, f) (n=3, one-way ANOVA with multiple comparisons).


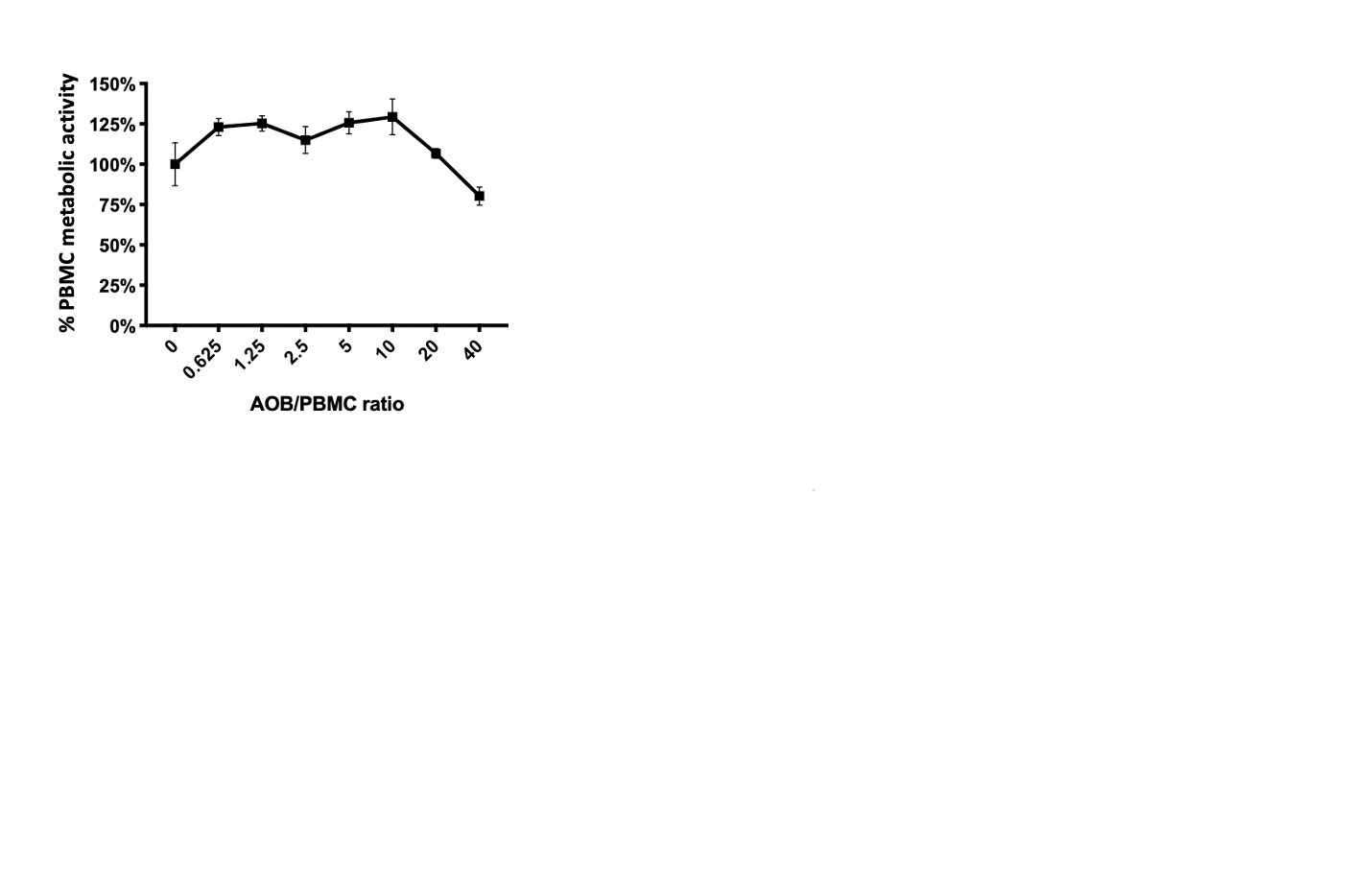


**Figure S4 – PBMC metabolic activity in presence of various AOB/PBMC ratios**

PBMC metabolic activity measured by WST-1 as an indicator of cell viability in samples collected 72h after treatment (n=3).


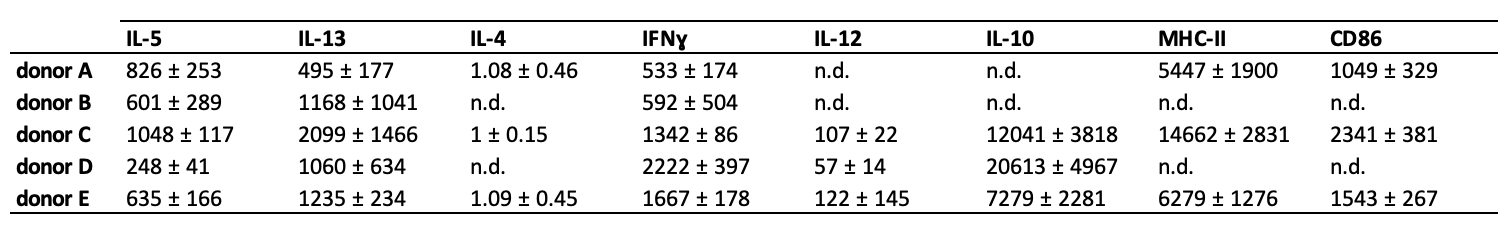


***Table S1 – Levels of cytokines and dendritic cell markers measured in the baseline Th2 stimulation condition for all donors evaluated in the study.***

*Data show the average +/- standard deviation of n=3-18 replicates. Cytokines levels are expressed in pg/mL and MHC-II & CD86 in mean fluorescence intensity (MFI).*
